# Supplementary material for: Asynchronous magnetic resonance elastography: Shear wave speed reconstruction using noise correlation of incoherent waves
Source: Magn Reson Med. 2022 Oct 27;89(3):990–1001. doi: 10.1002/mrm.29502 (PMC9792433; doi:10.1002/mrm.29502)
Supplement: Supplementary file 1 — DATA S1 MATLAB code used to generate the numerical simulations of Figure 1 [file MRM-89-990-s002.zip › k-Wave/helpfiles/kWaveGrid.html]

kWaveGrid :: Functions (k-Wave)


# kWaveGrid

Class definition for k-Wave grid.

## Syntax

```
kgrid = kWaveGrid(Nx, dx)
kgrid = kWaveGrid(Nx, dx, Ny, dy)
kgrid = kWaveGrid(Nx, dx, Ny, dy, Nz, dz)
```

## Description

`kWaveGrid` is the grid class used across the k-Wave Toolbox. An object of the `kWaveGrid` class contains the grid coordinates and wavenumber matrices used within the simulation and reconstruction functions in k-Wave. The grid matrices are indexed as: (x, 1) in 1D; (x, y) in 2D; and (x, y, z) in 3D. The grid is assumed to be a regularly spaced Cartesian grid, with grid spacing given by `dx`, `dy`, `dz` (typically the grid spacing in each direction is constant).

## Inputs

|  |  |
| --- | --- |
| `Nx, Ny, Nz` | number of grid points in each Cartesian direction |
| `dx, dy, dz` | grid point spacing in each Cartesian direction [m] |

## Outputs

|  |  |
| --- | --- |
| `kgrid` | `kWaveGrid` object used by the simulation and reconstructions functions within k-Wave |

## Dynamic Properties

Properties which can be queried or modified after the object is created.

| Fieldname | Description |
| --- | --- |
| `kgrid.Nt` | number of time steps (default = 'auto') |
| `kgrid.dt` | time step [s] (default = 'auto') |
| `kgrid.t_array` | evenly spaced array of time values [s] (default = 'auto') |

Note, `t_array` is a derived property, thus changing `Nt` or `dt` will also change `t_array`, and modifying `t_array` will update `Nt` and `dt`.

## Static Properties

Properties which can be queried, but not modified, after the object is created.

| Fieldname | Description |
| --- | --- |
| `kgrid.k` | `Nx` x `Ny` x `Nz` grid of the scalar wavenumber, where `k = sqrt(kx.^2 + ky.^2 + kz.^2)` [rad/m] |
| `kgrid.k_max` | maximum wavenumber (spatial frequency) supported by the grid [rad/m] |
| `kgrid.dim` | number of spatial dimensions |
| `kgrid.total_grid_points` | total number of grid points (equal to `Nx*Ny*Nz`) |
| `kgrid.highest_prime_factors` | 3 x 1 vector of the highest prime factor in each direction |

And for each spatial dimension x, y, z:

| Fieldname | Description |
| --- | --- |
| `kgrid.Nx` | number of grid points in the x-direction |
| `kgrid.dx` | grid point spacing in the x-direction [m] |
| `kgrid.x_vec` | `Nx` x 1 vector of the grid coordinates in the x-direction [m] |
| `kgrid.x` | `Nx` x `Ny` x `Nz` grid containing repeated copies of the grid coordinates in the x-direction [m] |
| `kgrid.x_size` | length of grid dimension in the x-direction [m] |
| `kgrid.kx_vec` | `Nx` x 1 vector of the wavenumber components in the x-direction [rad/m] |
| `kgrid.kx` | `Nx` x `Ny` x `Nz` grid containing repeated copies of the wavenumber components in the x-direction [rad/m] |
| `kgrid.kx_max` | maximum wavenumber (spatial frequency) supported by the grid in the x-direction [rad/m] |

## Methods: setTime

`setTime` sets `Nt` and `dt` to the input values. The syntax is:

```
kgrid.setTime(Nt, dt)
```

## Methods: makeTime

`makeTime` automatically specifies `Nt`, `dt`, and `t_array` based on the Courant-Friedrichs-Lewy (CFL) number and the grid size. The sytax is:

```
kgrid.makeTime(sound_speed)
kgrid.makeTime(sound_speed, cfl)
kgrid.makeTime(sound_speed, cfl, t_end)
kgrid.makeTime(sound_speed, [], t_end)
```

where the inputs are given by

```
sound_speed - sound speed in the medium [m/s]
cfl         - CFL number (default = 0.3)
t_end       - simulation time [s]
```

The time-step `dt` is chosen based on the CFL number (the default is 0.3), where `dt = cfl * dx / sound_speed`. If `t_end` is not specified, the number of time-steps is chosen based on the time it takes to travel from one corner of the grid to the geometrically opposite corner. Note, if `sound_speed` is given as a matrix, the calculation for `dt` is based on the maximum value, and the calculation for `t_end` based on the minimum value.

## Methods: Discrete Trigonometric Transforms

Objects of the `kWaveGrid` class also have the following methods to return the wavenumbers for use with discrete sine and cosine transforms. The syntax is:

```
k      = kgrid.k_dtt(dtt_type)
kx_vec = kgrid.kx_vec_dtt(dtt_type)
ky_vec = kgrid.ky_vec_dtt(dtt_type)
kz_vec = kgrid.kz_vec_dtt(dtt_type)
```

Here `dtt_type` is the type of discrete trigonometric transform (this determines the assumed symmetry of the input function), where:

```
1 -> DCT-I    WSWS (W: Whole Sample, S: Symmetric)
2 -> DCT-II   HSHS 
3 -> DCT-III  WSWA
4 -> DCT-IV   HSHA
5 -> DST-I    WAWA
6 -> DST-II   HAHA (H: Half Sample, A: Antisymmetric)
7 -> DST-III  WAWS
8 -> DST-IV   HAHS
```

These methods can also return the implied periodic length of the grid (denoted `M`) given the grid size and the `dtt_type`, e.g.,

```
[kx_vec, Mx] = kgrid.kx_vec_dtt(1);
[k, M]       = kgrid.k_dtt(1);
```

The method `.k_dtt` returns the product of the implied length in each dimension, e.g., in 2D, `M = Mx*My`. This is used for scaling the inverse trigonometric transforms.

## Example

Running `kgrid = kWaveGrid(128, 0.1, 128, 0.1)` at the command line will return:

```
kgrid = kWaveGrid(128, 0.1, 128, 0.1)

kgrid = 

  kWaveGrid with properties:

                       Nx: 128
                       Ny: 128
                       Nz: 0
                       dx: 0.1000
                       dy: 0.1000
                       dz: 0
                   kx_vec: [128x1 double]
                   ky_vec: [128x1 double]
                   kz_vec: 0
                        k: [128x128 double]
                   kx_max: 31.4159
                   ky_max: 31.4159
                   kz_max: 0
                    k_max: 31.4159
                      dim: 2
               nonuniform: 0
                       dt: 'auto'
                       Nt: 'auto'
                    x_vec: [128x1 double]
                    y_vec: [128x1 double]
                    z_vec: 0
                        x: [128x128 double]
                        y: [128x128 double]
                        z: 0
                       kx: [128x128 double]
                       ky: [128x128 double]
                       kz: 0
                   x_size: 12.8000
                   z_size: 0
                   y_size: 12.8000
        total_grid_points: 16384
    highest_prime_factors: [2 2 0]
                  t_array: 'auto'
```

## See Also

`kspaceFirstOrder1D`, `kspaceFirstOrder2D`, `kspaceFirstOrder3D`, `kspaceSecondOrder`, `pstdElastic2D`, `pstdElastic3D`
